# Validation of routine lymphatic filariasis morbidity surveillance in the Upper West Region, Ghana

**DOI:** 10.1371/journal.pgph.0004336

**Published:** 2025-04-02

**Authors:** Hope Simpson, Kofi Agyabeng, Bright Alomatu, Ernest Mensah, Solomon Atinbire, Melissa Edmiston, Kofi Asemanyi-Mensah, Joseph Opare, Rachel L. Pullan

**Affiliations:** 1 Department of Disease Control, London School of Hygiene and Tropical Medicine, London, United Kingdom; 2 Global Health and Infection Department, Brighton and Sussex Medical School, Falmer, United Kingdom; 3 Biostatistics Department, School of Global Public Health, New York University, New York, New York, United States of America; 4 Ghana Health Service, Neglected Tropical Diseases Programme, Accra, Ghana; 5 American Leprosy Missions, Accra, Ghana; 6 American Leprosy Missions, Greenville, South Carolina, United States of America; T D Medical College, INDIA

## Abstract

In Ghana and other countries pursuing elimination of lymphatic filariasis (LF), suspect cases of lymphoedema and hydrocele are routinely enumerated by community health workers (CHWs) during mass drug administration (MDA). These data, in addition to cases captured through the routine out-patient department are used for burden estimation and health service planning. To date there has been no systematic evaluation of the reliability of these data in Ghana. In December 2022, we conducted a cross-sectional survey of LF morbidity in two evaluation units in the Upper West Region of Ghana, including 19,180 participants. Participants with swelling affecting the scrotum or limbs were examined by clinicians to confirm whether symptoms were due to LF. Participants were asked whether their household had been visited by a CHW during the previous MDA. Suspect cases were asked whether they had reported their condition to a CHW or sought care elsewhere. We estimated the prevalence of each condition according to survey data and pre-existing routine data collected by CHWs and compared estimates. Lymphoedema prevalence rates were 87.3 and 61.2 per 10,000 in the two evaluation units, and hydrocele prevalence rates were 111.3 and 65.3 per 10,000 males. Routine enumeration underestimated lymphoedema prevalence by 81% in both cases, and underestimated hydrocele prevalence by 41%–52%. Nearly all households were visited during the previous MDA, but only 60.7% of lymphoedema and 28.3% of hydrocele cases had reported symptoms. 61.8% of lymphoedema and 42.9% of hydrocele cases had sought care from health facilities. Routine surveillance underestimates the prevalence of LF morbidity in the study area. Process modifications, including re-training of CHWs and health workers should be considered to improve data for service planning and validation of LF elimination. Anticipating cessation of MDA, continuous health service delivery, with periodic coverage evaluation, should be prioritised to strengthen passive surveillance.

## Introduction

Lymphatic filariasis is a parasitic mosquito-borne neglected tropical disease (NTD), with a widespread distribution across rainforest, grassland, and semi-arid climate regions of Africa, south and southeast Asia, and small parts of central America [1]. Since 2000, infection prevalence has declined in endemic areas and transmission has been interrupted in some countries, largely due to mass drug administration (MDA) of preventive chemotherapy (PC) [1,2]. Meanwhile, millions of people worldwide remain affected by its debilitating chronic manifestations: hydrocele, caused by the accumulation of peritoneal fluid inside the scrotal sac, and lymphedema, caused by accumulation of lymph fluid in the soft tissues which usually affects the limbs or breasts [3]. Lymphedema is associated with acute inflammatory episodes, which severely impact the quality of life and economic productivity of people affected [4,5]

Efforts to eliminate LF are coordinated by the Global Programme to Eliminate Lymphatic Filariasis (GPELF) [6], and implemented at national level by various NTD programmes (NTDPs). Validation of LF elimination depends on the sustained reduction of infection prevalence to levels at which transmission cannot be maintained, the availability of morbidity management and disability prevention (MMDP) services in health facilities in endemic areas, and documentation of lymphedema and hydrocoele case numbers at district level [7]. Morbidity burden estimates are required in order to appropriately plan MMDP services. According to the GPELF, essential MMDP for lymphoedema includes management of acute attacks and provision of information to support self-care, which should be available in at least one facility in each endemic or previously endemic district. For hydrocele, surgical services for hydrocelectomy should be available to cases in endemic or previously endemic districts. Since 2000, the NTDP in Ghana has made good progress towards these goals. As of 2024, LF transmission has been interrupted in 109 of 114 endemic districts [8]. Additionally, MMDP services were being integrated into all public health facilities across the country [9], and enumeration of LF morbidity (LFM) had been, or was being, conducted during MDA activities.

As in other countries currently enumerating cases of LFM, the NTDP in Ghana collects data on suspect cases through community drug distributors (CDDs) during MDA [10,11]. In Ghana, CDDs record the number of reported or observed arm, leg, or breast swellings (suspected lymphedema) and scrotal swellings (suspected hydrocele), and cases are validated by health workers in health facilities. CDDs typically only record new cases of LFM in each MDA round, and rely on their familiarity with the local community to distinguish new cases from those that they have previously recorded. Challenges in the routine surveillance of LFM have been noted in Ghana and in other countries. These include under-estimation of morbidity burden [12] and misdiagnosis of hydrocele, most commonly as hernia [13]. Such shortcomings are concerning because planning on the basis of incomplete data would result in insufficient resource allocation and under-provision of services to patients. Case-level data on hydrocele is also required to identify those eligible for surgery and to track the backlog of cases once hydrocelectomy services are rolled out. While estimates of LFM prevalence are widely available in Ghana, their reliability is largely unknown.

In this study, we aimed to provide robust prevalence estimates of LF morbidity (hydrocele and lymphedema) in two evaluation units in Ghana and to evaluate the reliability of routine morbidity data collected during MDA campaigns. We also aimed to investigate the proportion of survey cases already known to the formal health system (whether from reporting to a CDD or to a health facility), and predictors of household reporting of cases to CDDs.

## Methods

### Ethics statement

All participants were provided with an information sheet written in English, which was explained to them in English or their own language by a member of the field team or a translator if they could not read it. Adult participants (aged 18 years and older) provided written informed consent, and child participants (aged 15-17 years old) provided written assent with informed consent provided by a parent or guardian. The study was approved by Ethical Review Boards of The London School of Hygiene and Tropical Medicine, UK (Ref 28047) and the Ghana Health Service (Ref 016/09/22). Additionally, the Health Director for the Upper West Region provided written approval for the study. In each community surveyed, data collectors met with the community leader(s) at least a week before the survey dates to obtain verbal permission to work within the community.

### Study area

**Fig 1:**
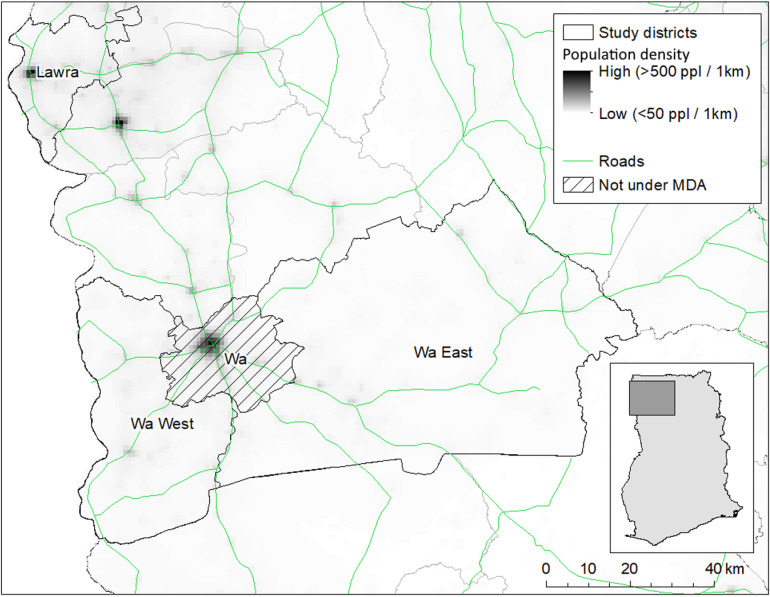
Map of the study area in the Upper West Region of Ghana. Country and district boundary shapefiles from the Database of Global Administrative Areas (GADM, v4.1) https://gadm.org/download_country.html [15], freely available for non-commercial use https://gadm.org/license.html. Roads shapefile from the Open Streetmap Project (OSM) [16], population density data from Worldpop [17].

The study area covered four districts in the Upper West Region (UWR) of Ghana (area: 18,476 km^2^; population: 901,502 [14]), in the north west of the country bordering Burkina Faso (Fig 1). The region lies in the dry Guinea Savannah Ecological zone and has a sub-Sahelian climate. The UWR has recorded the highest numbers of LF morbidity cases in Ghana, and despite several years of MDA and vector control measures, some areas continue to serve as transmission hotspots. At the time of the survey, eight of the 11 districts had interrupted transmission, while Wa West, Wa East and Lawra remained endemic. Medical officers in the region had been trained in hydrocelectomy using the WHO modified method. Lymphoedema management training commenced in 2019, but since then there has been a high attrition rate of health workers in the region.

The study districts make up two programmatic evaluation units (EUs): EU1 comprises the districts Wa East and Lawra and EU2 comprises Wa West and Wa Municipal (Table 1). These districts are indicated to have high levels of LF-morbidity based on routine community-based case finding, and have not participated in a previous mop-up survey for hydrocele and lymphedema. Wa Muncipal District passed TAS1 and has been classified as non-endemic for LF since 2015, and was no longer under MDA. As such, there had been no estimation of LFM case numbers since 2015.

**Table 1:** Total and stratified populations and pre-recorded (historical) numbers of lymphatic filariasis morbidity cases.

Evaluation Unit (and constituent districts)	Population^1^			Registered LFM cases (2022)^2^		
**Total**	**15 years**+	**Male 15 years**+	**All**	**Lymphedema**	**Hydrocele**
Lawra	67,536	40,451	18,572	88	47	41
Wa East	93,561	50,639	25,261	187	23	164
**EU1 (Wa East and Lawra)**	**161,097**	**91,090**	**43,834**	**275**	**70**	**205**
Wa Municipal	205,287	130,446	67,539	134	7	127
Wa West	99,187	54,870	25,987	290	98	192
**EU2 (Wa Municipal and Wa West)**	**304,474**	**185,316**	**93,526**	**424**	**105**	**319**

^1^Total population estimate used by the Ghana Health Service for programmatic purposes. Sub-population estimates based on stratified estimates from the National Census of Ghana 2010 [18].

^2^LFM = lymphatic filariasis morbidity, registered by community drug distributors during screening campaigns. Data from EU1 is the cumulative total number of cases registered up to 2022; data from Wa West is equivalent; data from Wa Municipal is from 2015.

### Study design

This was a cross-sectional population-based prevalence survey, with recruitment from December 9-21^st^, 2022. Primary sampling units (PSU) were defined as communities, the smallest locally recognized ‘administrative’ area with population data available. In the absence of a household sampling frame, we used a compact segment sampling design derived from the “modified segment design” option described in the manual for UNICEF’s Multiple-Indicator Cluster Surveys [19] and Turner et al. [20]. This is similar to the methodology described in WHO’s Coverage Evaluation Survey guidelines [21]. Compact-segment sampling has been demonstrated to give comparable results compared to random-walk methods, but reduces the risk of subjective selection of households [22].

Clusters were divided into segments of ~50 households and then one segment (or two segments for communities with more than 400 households) was randomly selected. Communities comprising fewer than 100 households were not segmented but surveyed exhaustively. Within selected segments, all households were visited, and all individuals aged 15 years and above residing in the household were invited to participate.

### Sample size calculation

We used a standard sample size calculation [23], assuming a prevalence of 5 cases of LFM per 1,000 population based on a previous survey conducted in Côte d’Ivoire [24], a design effect of 5.5, and a participation rate of 85%. We estimated that 19,787 people would need to be examined to estimate prevalence with an absolute error margin of 2.5 per 1,000 and a confidence level of 95%.

The sample size was divided into clusters of 100 people (approximately 50 households assuming 2 adults per household would be available for inclusion). Highly urban areas, defined as those with >10,000 residents were excluded, as such areas are not included in MDA distribution within the study area. The number of PSUs selected per district was proportional to district population size (higher numbers of clusters were sampled in larger districts). A list of communities with population sizes used by the district health team for planning purposes was used as the sampling frame. After exclusion of highly urban communities, the community population data indicated a population of 55,216 in Lawra, 93,974 in Wa East, 202,107 in Wa Municipal and 94,845 in Wa West. The number of clusters selected was 22 in Lawra, 38 in Wa East, 82 in Wa Municipal and 38 in Wa West.

### Study procedures

The data collection team consisted of six teams of four, each with two data collectors (local public health officers), one clinician, and one team lead. All data collectors received training on COVID-19 precautions, survey procedures, use of the open data kit (ODK)-based mobile data collection tool, informed consent, and the clinical diagnosis of lymphedema and hydrocele.

In consenting households, the household head was interviewed to collect basic information on proxies of household wealth, access to water and sanitation, and whether the household had been included in recent NTD control activities. Eligible participants were people aged 15 years and over. Consenting participants underwent a brief clinical examination for possible lymphedema, and males were asked to report any signs of scrotum swelling. Those with signs of swelling (observed or reported) were then examined in detail by a clinician, and interviewed to collect further details, including history of care seeking, diagnosis and previous treatment and health advice received. To limit the transmission risk of COVID and other respiratory infections, interviews and limb examinations were conducted outside participants’ homes, unless participants preferred otherwise. Genital examinations were conducted in a private space inside participants’ homes.

The case definition for clinically confirmed filarial lymphoedema was: swelling of limb or breast, in a person aged 15 years or older, present for at least a year but not since birth, and not due to other causes including pregnancy, injury, insect bite or allergy, leprosy, bacterial skin infection, heart disease and other systemic conditions, cancer, or lymph node removal. The case definition for clinically confirmed hydrocele was: a discrete, nontender mass around the testes, not present since birth and not present for less than 24 hours, and not explained by an inguinal hernia, variocele or tumour. All cases identified were confirmed through clinical examination by a clinician.

Lymphedema patients identified were referred to local health facilities for management. Cases of hydrocele were included in surgery lists to be invited to surgery camps to be established based on the results of the survey.

### Data analysis

#### Prevalence estimation.

Evaluation unit-level prevalence estimates and expected case numbers of lymphedema, hydrocele, leg swellings, scrotal swellings, and all LF morbidity were calculated using the *survey* package in R [25]. Lymphedema and leg swelling prevalence rates were calculated by dividing the number of cases by the number examined; hydrocele and scrotal swelling prevalence rates by dividing the number of cases by the number of males examined. Estimates were adjusted for survey design and post-stratified for age and gender using stratified populations the Ghana 2010 Census [26].

#### Reliability of pre-existing data on lymphatic filariasis morbidity.

Pre-existing data on LFM cases identified by CDDs (S1 Table) were used to estimate the crude prevalence of suspected hydrocele, lymphedema, and all LFM at EU level. For Wa Municipal, there had only been one round of community-based morbidity screening (in 2015), so the number of cases recorded in that year was used. For the other districts, LFM prevalence estimates were calculated from the most recent year available. Each year of data represents a cumulative total of recorded cases, as CDDs only register cases not previously recorded in each annual campaign.

Population denominators for calculation of crude prevalence rates of suspect LFM detected by CDDs were derived from GHS estimates of the total population at district level provided by the NTDP (Table 1) and age and gender estimates from the 2010 national census [18]. Prevalence estimates from the CBS and the survey were compared using prevalence ratios with confidence intervals.

#### Household inclusion in morbidity enumeration.

In each district surveyed and currently undergoing MDA (Lawra, Wa East and Wa West), we calculated the proportion of households surveyed which i) reported being visited by a CHW/CDD during the last round of MDA; ii) were aware that CHWs/CDDs were screening for swelling at the same time.

#### Cases known to the health system.

For districts still undergoing MDA, we calculated the proportion of cases which had been reported to CHWs/CDDs. In all study districts, we calculated the proportion of all cases who had sought care for the condition, classifying by the type of care sought (health facilities,; alternative providers, including traditional healers and herbalists; and other providers, including pharmacists and drug sellers).

Among households with cases of LFM confirmed in the survey, within districts still undergoing MDA, we explored predictors of reporting cases to CHWs using a mixed-effects generalized linear model (binomial distribution). The model outcome was reported notification of a case of lymphedema or hydrocele to CHWs. District and subdistrict were included as fixed effects. We explored potential correlates from the survey: household mobile phone ownership, the number of adults and total size of the household, household head primary language (majority or other), household head gender, education of the household head (none or any), and type of morbidity within the household (any lymphoedema cases or none and any hydrocele cases or none). Continuous variables were mean-centred and scaled. Sociodemographic variables collected in the survey but not selected as potential predictors of reporting to CHWs used to create a multi-dimensional indicator of household socioeconomic status (SES) with three levels (lower, middle, and upper). We first conducted a univariate analysis to determine crude relationships between each predictor and the outcome. We included variables associated with a likelihood ratio test (LRT) p-value of 0.2 or lower in a multivariable model which was refined through backwards stepwise selection. For this, we dropped variables in order of their effect size and selected the best-fitting model, evaluated by reduction in the Akaike Information Criterion (AIC).

### Deviations from protocol

In the protocol provided to ethical review boards, we stated that we would use filariasis test strips to assist with diagnosis of lymphatic filariasis morbidity. Due to supply issues, it was not possible to obtain these diagnostics, so clinical diagnosis alone was used.

### Inclusivity in global research

Additional information regarding the ethical, cultural, and scientific considerations specific to inclusivity in global research is included in the Supporting Information (S2 Checklist).

## Results

### Basic characteristics of participants

Out of 8,961 households visited, the household head refused to participate in 17 (0.19%). Of 19,210 people invited to participate, 30 (0.16%) did not consent. Fieldworkers screened a total of 19,180 participants (including 8,129 males; 42.4% of all participants) from 8,303 households in 180 communities. Examination for lymphoedema was not conducted for 10 people (0.05%). Of those who reported scrotal swellings, all agreed to undergo clinical examination. Key characteristics of participants are shown in Table 2. An anonymized version of the analysis dataset is available from the Harvard Dataverse [27].

**Table 2:** Characteristics of participants and households.

Characteristic	n (%)
**District**	
Lawra	2,267 (11.8%)
Wa East	3,525 (18.4%)
Wa Municipal	9,192 (47.9%)
Wa West	4,196 (21.9%)
**Gender**	
Female	11,051 (57.6%)
Male	8,129 (42.4%)
**Age**	
15–25	5,223 (27.2%)
25–35	4,358 (22.7%)
35–45	3,380 (17.6%)
45–55	2,680 (14%)
55–65	1,577 (8.2%)
65–125	1,962 (10.2%)
**Household electricity supply**	
No	6,644 (34.6%)
Yes	12,531 (65.3%)
Don’t know/ refused to answer	5 (<0.01%)
**Household improved water supply**	
No	790 (4.1%)
Yes	18,389 (95.9%)
Don’t know/ refused to answer	1 (<0.01%)
**Household motorised vehicle**	
No	9,665 (50.4%)
Yes	9,491 (49.5%)
Don’t know/ refused to answer	24 (0.1%)
**Household mobile phone**	
None	2,538 (13.2%)
Smartphone	7,553 (39.4%)
Basic mobile phone	9,086 (47.4%)
Don’t know/refused to answer	3 (<0.01%)

### Prevalence of lymphatic filariasis morbidity

Among 97 cases of leg swellings, 74 were clinically confirmed as filarial lymphedema, and among 121 cases of scrotal swellings, 77 were confirmed as filarial hydrocele (Table 3).

**Table 3:** Comparison of lymphatic morbidity prevalence estimates from the community-based screening and population-based survey.

	Community-based screening				Population-based survey					*Prev. ratio* *(screen to survey)*
	Population^1^ (N)	Suspect cases (n)	Crude prev^2^.	*(95% CI)*	Examined (N)	Confirmed cases (n)	Weighted			
							Estimated cases	Prev^1^.	*(95% CI)*	
**Wa East (2022) and Lawra (2022)**										
LF morbidity	91,090	275	30.2	(26.7–34.0)	5,789	59	795	87.3	(82.0–92.7)	0.35 (0.30–0.39)
All limb swellings	91,090	70	7.7	(6.0–9.7)	5,789	45	545	59.8	(55.4–64.4)	0.13 (0.10–0.16)
LF lymphedema						30	378	41.4	(38.3–44.8)	0.19 (0.14–0.24)
All scrotum swellings	43,833	205	46.8	(40.6–53.6)	2,582	55	854	194.8	(183.1–206.8)	0.24 (0.20–0.28)
LF hydrocele						34	496	113.1	(104.7–121.8)	0.41 (0.35–0.49)
**Wa West (2022) and Wa Municipal (2015)**										
LF morbidity	185,316	424	22.9	(20.8–25.2)	13,382	88	1157	61.2	(58.0–64.5)	0.37 (0.33–0.42)
All limb swellings	185,316	105	5.7	(4.6–6.9)	13,382	52	650	34.4	(32.4–36.5)	0.16 (0.13–0.20)
LF lymphedema						45	552	29.2	(27.4–31.1)	0.19 (0.16–0.24)
All scrotum swellings	93,526	319	34.1	(30.5–38.1)	5,529	66	1035	110.6	(104.4–117.1)	0.31 (0.27–0.35)
LF hydrocele						43	611	65.3	(60.9–69.8)	0.52 (0.45–0.60)

^1^Population aged 15 years and older, based on population estimates used by the Ghana Health Service for programmatic purposes, adjusted by the proportion of population>=15 years and the proportion of males from the Ghana 2010 National Census [18].

^2^Prev = prevalence per 10,000 population. Hydrocele prevalence is calculated among males only.

Adjusting for survey design and non-response bias, the prevalence of LF morbidity (i.e., filarial lymphedema and filarial hydrocele) in EU1 (Wa East and Lawra) was 87.3 per 10,000 population (95% confidence interval [CI] 82.0–92.7 per 10,000), and that in EU2 (Wa West and Wa Municipal) was 61.2 (95% CI 58.0–64.5) per 10,000. The prevalence of filarial lymphedema in EU1 was 41.4 (95% CI 38.3–44.8) per 10,000, and that in EU2 was 29.2 (95% CI 27.4–31.1) per 10,000. Among males, the prevalence of filarial hydrocele was 113.1 (95% CI 104.7–121.8) per 10,000 in EU1, and 65.3 (95% CI 60.9–69.8) per 10,000 in EU2. The total prevalence of scrotal swellings was 183.1 (95% CI 183.1–206.8) per 10,000 males in EU1 and 110.6 (95% CI 104.4–117.1) per 10,000 males in EU2.

### Reliability of pre-existing data on lymphatic filariasis morbidity

To estimate the reliability of pre-existing morbidity data, we calculated screen to survey prevalence ratios, and considered the pre-existing data to be reliable if confidence intervals included 1. All outcomes were underestimated. The level of underestimation was variable with respect to different outcomes, but relatively consistent between the two EUs. Total LFM was underestimated by approximately 65% in both IUs, with prevalence ratios of 0.35 (95% CI 0.30–0.39) in EU1 and 0.37 in EU2 (95% CI 0.33–0.42). In both EUs, lymphedema prevalence was underestimated by 81%, with a prevalence ratio of 0.19 (95% CI 0.14–0.24) in EU1, and 0.19 (95% CI 0.16–0.24) in EU2. Hydrocele prevalence was underestimated to a lesser degree, with a prevalence ratio of 0.41 (0.35–0.41) in EU2, 0.52 in EU2 (95% CI 0.45–0.60).

### Household inclusion in morbidity enumeration

In the districts still undergoing MDA (including 4,290 surveyed households), reported household coverage of MDA was very high–over 96% of households reported having been visited by a CDD in all districts. Of the households which had been visited during MDA, 71.3% of those in Lawra, and over 90% of those in Wa East and Wa West were aware that CHWs were also screening residents for swelling during the MDA activity.

### Cases known to the health system

In districts undergoing MDA, 15 of 53 cases of hydrocele (28.3%) and 37/61 cases of lymphedema (60.7%) informed researchers they had reported their condition to CHWs in the last twelve months (Table 4). In these same districts, 23/53 cases of hydrocele (43.4%), and 36/61 cases of lymphedema (59.0%) had sought care at a health facility (a government CHPS or health center or a Christian health facility), while 5 cases of hydrocele (6.5%) and 20 of lymphedema (27.6%) had sought care from alternative providers (traditional healers and herbalists). In Wa Municipal, the only district surveyed no longer under MDA, 10/24 cases of hydrocele (41.7%) and 11/15 cases of lymphedema (73.3%) had sought care in health facilities.

**Table 4:** Inclusion of lymphatic filariasis morbidity cases in community-based screening.

	N	HH visited by CHW		HH reported case to CHW^1^		Sought care							
**Health facility** ^2^		**Alternative** ^3^		**Other** ^4^		**None**	
		n	*(%)*	n	*(%)*	n	*(%)*	n	*(%)*	n	*(%)*	n	*(%)*
**Lawra**													
Hydrocele	13	13	*(100)*	6	*(46.2)*	8	*(61.5)*	0	*(0.0)*	0	*(0.0)*	5	*(38.5)*
Lymphedema	20	19	*(95)*	13	*(65.0)*	18	*(90.0)*	1	*(5.0)*	0	*(0.0)*	1	*(5.0)*
**Wa East**													
Hydrocele	21	21	*(100)*	7	*(33.3)*	6	*(28.6*	3	*(14.3)*	4	*(19.0)*	8	*(38.1)*
Lymphedema	10	10	*(100)*	8	*(80.0)*	3	*(30.0)*	4	*(40.0)*	0	*(0.0)*	3	*(30.0)*
**Wa West**													
Hydrocele	19	19	*(100)*	2	*(10.5)*	9	*(47.4)*	2	*(10.5)*	2	*(10.5*	6	*(31.6)*
Lymphedema	31	31	*(100)*	16	*(51.6)*	15	*(48.4)*	15	*(48.4)*	0	*(0.0)*	1	*(3.2)*
**Total in districts with MDA**													
Hydrocele	53	53	*(100)*	15	*(28.3)*	23	*(43.4)*	5	*(9.4)*	6	*(11.3)*	19	*(35.8)*
Lymphedema	61	60	*(98.4)*	37	*(60.7)*	36	*(59.0)*	20	*(32.8)*	0	*(0.0)*	5	*(8.2)*
**Wa Municipal**													
Hydrocele	24	--		--		10	*(41.7)*	0	*(0.0)*	0	*(0.0)*	14	*(58.3)*
Lymphedema	15	--		--		11	*(73.3)*	1	*(6.7)*	0	*(0.0)*	3	*(20.0)*
**Total in study area**													
Hydrocele	77	53	*(68.8)*	15	*(19.5)*	33	*(42.9)*	5	*(6.5)*	6	*(7.8)*	33	*(42.9)*
Lymphedema	76	60	*(78.9)*	37	*(48.7)*	47	*(61.8)*	21	*(27.6)*	0	*(0.0)*	8	*(10.5)*

^1^Case occurred in a household which described reporting a case to CHWs in the last 12 months.

^2^Includes government community health planning and services (CHPS) facilities, government health facilities, and Christian health facilities

^3^Includes traditional healers and herbalists.

^4^Includes drug sellers and pharmacists and other types of care.

Among households with confirmed cases of LF morbidity located in districts undergoing MDA (n=108), those including lymphedema cases (n=61) compared to those with hydrocele cases only had 45% higher odds of reporting cases to the CHWs (aOR: 1.45, 95%CI: 1.23–1.71, p<0.001, Table 5). There was no evidence of a difference in reporting rates across any of the socio-economic factors explored, including primary language of the household head, education level of the household head, household size, or household socioeconomic category.

**Table 5:** Predictors of reporting of lymphatic filariasis morbidity cases by their households.

Predictors of reporting to CHWs	N surveyed	n reporting to CHW	Probability	Adjusted OR (95% CI)	LRT p-value
**Fixed Effects**
**Lymphedema cases**					<0.001
None (hydrocele only)	47	11	23.4		
One or more	61	37	60.7	1.45 (1.23-1.71)	
				**ICC**	
				community zones within districts	0.265
				community zones	0.014

Model adjusted for fixed effects of community zones nested within districts.

## Discussion

In this study, we conducted a population-based prevalence survey to evaluate the reliability of routine surveillance data collected by community health workers in four districts (two evaluation units) of Ghana. We found a high burden of lymphatic filariasis morbidity, which affected 87 and 61 people per 10,000 population (aged 15 years and older) in the two EUs respectively. The prevalence survey indicated that pre-existing data substantially underestimated the prevalence of both lymphedema and hydrocele, that its reliability varied between outcomes, but was similar across the two EUs. Previous care seeking varied between districts but was higher among lymphoedema cases compared to hydrocele cases.

We found that hydrocele and lymphedema were both underestimated by CHWs, and that the difference between CBS and prevalence survey estimates was more extreme for lymphoedema. Previous studies have indicated that LF morbidity screening during MDA tends to underestimate prevalence [12,24]. It is likely that this reflects a combination of challenges to achieving high coverage, reliable detection, and accurate reporting. While these may vary in importance between settings, evidence suggests that recording of morbidity cases during MDA is a particularly strong barrier [11,12]. For example, a study in Malawi indicated that, when asked, community-level health workers were able to list more than twice the number of lymphoedema cases they had recorded during recent MDA [12]. This study also showed that community-level health workers reliably identified both lymphoedema and hydrocele in Malawi, while CDDs in Ghana were able to reliably identify lymphoedema but commonly misdiagnosed cases of inguinal hernia as hydrocele [12]. Pilots of community-led reporting of LF morbidity by mobile phones in Malawi and Ghana demonstrated that during 5-14 days, CHWs were able to identify 2-14 times more cases of lymphoedema and hydrocele compared to the case numbers recorded during MDA [11]. In a study in Côte d’Ivoire, in which morbidity screening by CDDs was decoupled from MDA, CDDs provided accurate estimates of the prevalence of lymphoedema and all scrotal swellings but over-estimated hydrocele prevalence, apparently due to misdiagnosis of inguinal hernia [24].

We found that almost 100% of households in districts still undergoing MDA had been visited by a CHW during the most recent distribution activity, but that less than 50% of lymphoedema cases and less than 20% of hydrocele cases had reported their symptoms to CHWs. This could suggest either that CHWs did not consistently ask about symptoms, or that some cases were not willing to divulge them. These results are somewhat at odds with the prevalence ratios calculated for the CBS and survey results, which indicated that CHWs had identified around one fifth of the total burden of lymphoedema and 41-52% of the hydrocele burden. This inconsistency could indicate that CHWs did not record all cases of self-reported lymphoedema, and that hydrocele cases were over-diagnosed by CHWs.

Of the cases identified in this study, 62% of those affected by lymphoedema and 43% of those with hydrocele reported having sought care for their condition in a health facility. These were higher than the proportions who said they had reported their symptoms to a CHW during MDA, indicating that cases may be more willing to self-report to clinicians. Compared to districts still under MDA, there was no difference in the proportion of cases from Wa Municipal who had sought care from health facilities, suggesting that the cessation of morbidity surveillance had not been accompanied by changes in demand for services. However, while notable proportions of cases outside of Wa Municipal had sought care from alternative healthcare providers (traditional healers, herbalists, drug sellers and pharmacists), this was very rare in Wa Muncipal, where nearly all cases who had not been to a health facility had not sought any other form of care.

In the context of previous work, our findings (particularly the high proportion of households visited by CHWs) indicate that MDA could offer a suitable platform for LF morbidity burden estimation, with modifications to recording and training in case identification by CHWs. The low proportion of cases who had self-reported to a CHW indicates that training programmes should cover communication skills to encourage cases to report their symptoms. In this study, it was not possible to identify the cases previously recorded by CDDs, and so the characteristics of cases missed could not be analysed. However, previous studies have highlighted the importance of training community health workers to identify early-stage cases of lymphoedema, given that these have a high potential for successful management [11]. This could be achieved by training CDDs to ask participants about reversible swelling (which may not be visible at the time of examination) and history of ADLA episodes.

However, it is critical to bear in mind that MDA does not offer a sustainable platform for case identification and burden estimation as Ghana and other countries progress towards LF elimination. As of 2024, all of the four districts surveyed have been validated as having eliminated LF and are therefore no longer under morbidity surveillance. Looking forward, alternative options include case detection through passive surveillance, prevalence surveys, and morbidity censuses decoupled from MDA.

Passive surveillance appears to offer several advantages: firstly the costs of recording and reporting are much lower than actively searching for or surveying cases, secondly that diagnosis would be more reliable as it would be done by health workers, and thirdly that medical treatment and advice could be offered at the time of diagnosis. However, in this study, around half of the cases identified had sought care at health facilities, indicating that substantial increases in service delivery and uptake (as well as reliable recording and reporting) would be required to provide reasonably accurate burden estimates and identify a large majority of cases. The Ghana NTDP aim to train and equip all public health facilities for essential lymphoedema management would help support this, but continued investment will be required to improve and maintain service delivery at the levels required. Initiatives such as community engagement and awareness raising activities would also be needed to increase health service uptake. Even with such strengthening, passive reporting would remain fallible to systemic biases resulting from inequities in access to health services [10].

Cluster-based prevalence surveys, if well designed and implemented, enable robust prevalence estimation without exhaustive screening. These have been used to estimate the burden of LF morbidity at district level in Bangladesh and Liberia [28–30]. A similar approach is used to estimate the prevalence of late stage trachoma (trachomatous trichiasis; TT) in some settings, as validation of trachoma elimination requires evidence that TT is less than 0.2% in previously endemic EUs [31]. These typically cover 30 clusters per EU and have been found to have a median cost of USD 9,707 [32]. In Thailand, existing platforms such as post-elimination blood surveys are used to identify cases not known to the health system [33]. However, a disadvantage of using surveys to estimate burden is that this approach does not identify every case, as case numbers are estimated based on the prevalence in surveyed clusters.

In Bangladesh, a national morbidity census was conducted from 2013-16, covering 19 high endemic and 15 low endemic districts with a total population of 65M people [34]. In high endemic districts, data collectors (including community healthcare providers, health assistants and family welfare advisors) carried out an exhaustive case search. In low-endemic districts, a central team gathered records from health facilities, validated cases through household visits, and recorded additional cases identified in communities. The average district level costs were USD 12,952 in high endemic districts and USD 7,922 in low endemic districts. In contrast to cluster-based surveys, this approach could enable comprehensive case identification as well as burden estimation, as long as cases are recorded at individual level and not simply tallied.

Undertaken by local health staff and clinicians from the Ghana Health Service, the survey achieved high coverage of the target population and generated robust and reliable prevalence estimates of morbidity due to LF. It also contributed to local health system capacity by providing training in the diagnosis of LF morbidity and management of lymphoedema to local health workers.

This study has several limitations which should be considered when interpreting the results. We were limited to two EUs, which might not reflect other settings in the country. Given the high costs of surveys, it is unlikely to be feasible survey every EU in the country. We used a door-to-door approach and so may have missed people not at home, but attempted to correct for this bias by adjusting survey estimates by the underlying population structure. The population estimates used for this adjustment (from the 2010 national census) may be somewhat outdated, but to our knowledge remain the most reliable source of information about the demographic composition of the study population.

There are also limitations affecting the routine data we evaluated. The data from Wa Municipal included only one year’s worth of data, as surveillance has not been conducted since 2015, while data from the other districts was accumulated between 2015 and 2022. In Ghana, CDDs do not register every existing case of LFM each year, but only those not already known to them, relying on their familiarity with the local community to avoid re-registering the same people. As such, the surveillance data aggregates newly identified cases with those previously identified each year, and we cannot be sure that the district totals do not contain duplicates, or people deceased since registration. Moving towards case-based reporting in the future would improve the reliability of the morbidity registers, and would also support planning and monitoring of case management activities.

## Conclusions

We identified sizeable numbers affected by morbidity, including in the district which had successfully reduced the transmission of LF. A substantial proportion of cases were not accessing health services, despite previous capacity building and services delivery initiatives. The existing approach to LF morbidity surveillance underestimates the true burden of disease, indicating that modifications are required for reliable burden estimation. However, given that MDA will not be conducted indefinitely, alternative options should be considered. Continued investment in services, community engagement, and monitoring of inclusion (for example through periodic surveys) will be required to meet the needs of this population.

## Supporting information

S1 Table Pre-existing estimates of suspect lymphatic filariasis morbidity identified through screening by community drug distributors during mass drug administration in the four study districts of Ghana, 2015-2022. (DOCX)

S1 Checklist PLOS Inclusivity in global research questionnaire. (DOCX)
