# Supplementary material for: Validation of routine lymphatic filariasis morbidity surveillance in the Upper West Region, Ghana
Source: PLOS Glob Public Health. 2025 Apr 2;5(4):e0004336. doi: 10.1371/journal.pgph.0004336 (PMC11964249; doi:10.1371/journal.pgph.0004336)
Supplement: S1 Table — (DOCX) [file pgph.0004336.s001.docx]

Supplementary Table 1: Pre-existing estimates of suspect lymphatic filariasis morbidity identified through screening by community drug distributors during mass drug administration in the four study districts of Ghana, 2015- 2022.

| District | 2015 | | 2016 | | 2017 | | 2018 | | 2019 | | 2021 | | 2022 | | Estimated prevalence | |
| --- | --- | --- | --- | --- | --- | --- | --- | --- | --- | --- | --- | --- | --- | --- | --- | --- |
|  | HC | LE | HC | LE | HC | LE | HC | LE | HC | LE | HC | LE | HC | LE | HC | LE |
| Lawra | 55 | 34 | 67 | 37 | 61 | 38 | 58 | 35 | 88 | 29 | 48 | 51 | 41 | 47 | 22.1 | 11.6 |
| Wa East | 1 | 8 | 36 | 4 | 71 | 7 | 86 | 6 | 67 | 18 | 50 | 11 | 164 | 23 | 64.9 | 4.54 |
| Wa Municipal | 127 | 7 |  |  |  |  |  |  |  |  |  |  |  |  | 18.8 | 0.54 |
| Wa West | 570 | 96 | 153 | 71 | 176 | 79 | 178 | 78 | 199 | 79 | 174 | 87 | 192 | 98 | 73.9 | 17.9 |
| **Total** | **753** | **145** | **256** | **112** | **308** | **124** | **322** | **119** | **354** | **126** | **272** | **149** | **397** | **168** |  |  |

*HC= hydrocele; LE= lymphedema. There was no community based screening in 2020.*

Population denominators for calculation of crude prevalence rates of suspect lymphatic filariasis morbidity detected by community drug distributors were derived from Ghana Health Service estimates of the total population at district level provided by the Neglected Tropical Diseases Programme. As this data source only provided the total population (not stratified by age and gender) we estimated populations aged 15 years and older, and male populations aged 15 years and older, using proportions of total population within these categories from the 2010 national census (1).

***References***

1. Ghana Statistical Service. 2010 population & housing census: National analytical report: Ghana Statistics Service; 2013.
